# Falls among older adults in the South of Brazil: prevalence and determinants

**DOI:** 10.11606/S1518-8787.2018052000103

**Published:** 2018-02-07

**Authors:** Luna S Vieira, Ana Paula Gomes, Isabel O Bierhals, Simone Farías-Antúnez, Camila G Ribeiro, Vanessa I A Miranda, Bárbara H Lutz, Thiago G Barbosa-Silva, Natália P Lima, Andréa D Bertoldi, Elaine Tomasi

**Affiliations:** I Universidade Federal de Pelotas. Programa de Pós-Graduação em Epidemiologia. Pelotas, RS, Brasil; II Universidade Federal de Pelotas. Faculdade de Medicina. Departamento de Medicina Social. Pelotas, RS, Brasil

**Keywords:** Aged, Accidental Falls, Prevalence, Risk Factors, Cross-Sectional Studies, Idoso, Acidentes por Quedas, Prevalência, Fatores de Risco, Estudos Transversais

## Abstract

**OBJECTIVE:**

Evaluate the prevalence and the factors associated with the occurrence of falls among older adults.

**METHODS:**

A cross-sectional study with a representative sample of 1,451 elderly residents in the urban area of Pelotas, RS, in 2014. A descriptive analysis of the data was performed and the prevalence of falls in the last year was presented. The analysis of demographic, socioeconomic, behavioral and health factors associated with the outcome was performed using Poisson regression with adjustment for robust variance according to the hierarchical model. The variables were adjusted to each other within each level and for the higher level. Those with p ≤ 0.20 were maintained in the model for confounding control and those with p < 0.05 were considered to be associated with the outcome.

**RESULTS:**

The prevalence of falls among older adults in the last year was 28.1% (95%CI 25.9–30.5), and most occurred in the person’s own residence. Among the older adults who fell, 51.5% (95%CI 46.6–56.4) had a single fall and 12.1% (95%CI 8.9–15.3) had a fracture as a consequence, usually in the lower limbs. The prevalence of falls was higher in women, adults of advanced age, with lower income and schooling level, with functional incapacity for instrumental activities, and patients with diseases such as diabetes, heart disease, and arthritis.

**CONCLUSIONS:**

The occurrence of falls reached almost a third of the older adults, and the prevalence was higher in specific segments of the population in question. About 12% of the older adults who fell fractured some bone. The factors associated with the occurrence of falls identified in this study may guide measures aimed at prevention in the older adult population.

## RESUMO

**OBJETIVO:**

Avaliar a prevalência e os fatores associados à ocorrência de quedas em idosos.

**MÉTODOS:**

Estudo transversal com amostra representativa de 1.451 idosos residentes na zona urbana de Pelotas, RS, em 2014. Foi realizada análise descritiva dos dados e apresentada a prevalência de quedas no último ano. A análise de fatores demográficos, socioeconômicos, comportamentais e de saúde associados ao desfecho foi realizada por meio de regressão de Poisson com ajuste para variância robusta conforme modelo hierárquico. As variáveis foram ajustadas entre si dentro de cada nível e para as de nível superior. Aquelas com p ≤ 0,20 foram mantidas no modelo para controle de confusão e aquelas com p < 0,05 foram consideradas associadas ao desfecho.

**RESULTADOS:**

A prevalência de quedas em idosos no último ano foi de 28,1% (IC95% 25,9–30,5), e a maioria ocorreu na própria residência do idoso. Entre os idosos que sofreram queda, 51,5% (IC95% 46,6–56,4) tiveram uma única queda e 12,1% (IC95% 8,9–15,3) tiveram fratura como consequência, sendo a de membros inferiores a mais relatada. A prevalência de quedas foi maior em mulheres, idosos com idade avançada, de menor renda e escolaridade, com incapacidade funcional para atividades instrumentais e portadores de enfermidades como diabetes, doença cardíaca e artrite.

**CONCLUSÕES:**

A ocorrência de quedas atingiu quase um terço dos idosos, e a prevalência foi mais elevada em segmentos específicos da população em questão. Cerca de 12% dos idosos que caíram, fraturaram algum osso. Os fatores associados à ocorrência de quedas identificados neste estudo poderão nortear medidas que visem sua prevenção na população de idosos.

## INTRODUCTION

The rapid aging of the population in low- and middle-income countries results in a significant change in morbidity and mortality due to the increase in the prevalence of chronic non-communicable diseases^1^ ^,^ ^2^. In addition to increased comorbidities, the use of different medications, slow gait, and decreased visual acuity and hearing tend to generate a disabling process of the elderly over time^1^ ^,^ ^3^. As a consequence of these functional changes, there is an exponential increase in the occurrence of trauma due to external causes, especially falls.

According to the World Health Organization (WHO), a fall is the unintentional movement of the body to a level lower than the initial position, with the inability to correct it in a timely manner^4^. The fall is determined by multifactorial circumstances, in which intrinsic (physiological changes characteristic of aging, presence of morbidities, deficits in balance, vision, hearing or walking) and extrinsic (environmental risks due to poor lighting or slipperiness, risky behaviors, such as climbing chairs or stairs, and those related to daily activities) factors are involved. Most of the time, it results from the interaction between these factors^5^.

About 30% of older adults suffer falls in a one-year period in Brazil, according to the Ministry of Health^1^. Although falls are responsible for the increased risk of injury, emotional problems, and death in this population group, and therefore represents a serious public health problem, it does not receive due attention from Brazilian society^3,6–8^. Thus, evaluating the factors that most often put older adults at risk of falling can provide important information for the planning of public policies that aim to prevent the occurrence of this incident in this population.

The objective of this study was to estimate the prevalence of self-reported falls in older adults (60 years or older) in a medium-sized city in the southern region of Brazil, and to identify their main associated factors.

## METHODS

A cross-sectional population-based study in the urban area of Pelotas in 2014, with the objective of understanding the health characteristics of the population aged 60 years and over. The research was conducted by the graduate program in Epidemiology of the Universidade Federal de Pelotas through the research consortium of master’s students^9^.

The calculation of the sample size for the prevalence study considered a prevalence of falls in older adults of 34.8%^10^, a 95% confidence level, a margin of error of four percentage points, and a design effect of two. With an increase of 10% for losses, the minimum sample size required would be 1,198 older adults. The minimum sample required for the association study was estimated at 1,113 older adults, a result of the association between falls and gender, based on the following parameters: 95% confidence level, 80% power, 40/60 ratio between unexposed and exposed, fall prevalence of 26.5% among unexposed (male gender)^10^, prevalence ratio of 1.5, design effect of two, additional 10% for losses and refusals, and 15% for control of possible confounding factors.

The sampling process was carried out in two stages. Initially, the census tracts were ordered according to the average income of each inhabitant based on the census of the Brazilian Institute of Geography and Statistics (IBGE) of 2010^11^. The first stage of the sampling process comprised the random draw of the tracts, which resulted in 133 selected tracts. In the second stage, the selection of approximately 30 households in each tract was made through a systematic draw. At the end of the process, 4,123 households were selected for the survey. Based on a previous estimate of the same census, which found 0.43 older adult/domicile, we expected to find at least 12 older adults people per tract, which would result in a sample of approximately 1,700 older adults (all the older adults of the household were included in the study). This number was considered adequate for the outcome, since, according to the sample calculation, a minimum sample of 1,198 older adults would be necessary.

The data collection was performed from January to August 2014 in the household of the older adult, by trained interviewers, through a questionnaire previously tested and built in a digital format, which was applied with the help of netbooks.

The outcome was the prevalence of falls in the older adult in the last year, investigated by the following question: “Have you fallen down from <month in the previous year> to today?”. Older adults who reported an affirmative answer had their past-year history of falls investigated by two additional questions about the number of falls that occurred and whether a limb fractured because of the fall.

Demographic characteristics examined as independent variables: gender (female or male), age (categorized as 60–69, 70–79 and 80 years old or more) and marital status (with partner or without partner). The socioeconomic characteristics were classified according to an instrument of the Brazilian Association of Research Companies (ABEP)^11^ and categorized into classes A/B, C, D/E), schooling level in complete years of study (none, 1 to 3, 4 to 7, 8 to 10, 12 or more), currently employed (no or yes). Regarding health variables, the following morbidities were studied: systemic arterial hypertension, diabetes, heart disease, arthritis, osteoporosis, Parkinson’s disease, nephropathy, glaucoma, and emphysema. We also investigated the occurrence of stroke at some point in life. Considering the presence of these diseases, a morbidity score was created, categorized as “none”, “1”, “2” or “≥ 3” morbidities.

The presence of sarcopenia was assessed according to the criteria proposed by the European Working Group on Sarcopenia in Older People^12^. The loss of muscle mass was determined by measuring with an inextensible tape (Cescorf, Brazil) the circumference of the calves of the older adult, based on cut-off points established in the population studied (≤ 34 cm for men and ≤ 33 cm for women were indicative loss of muscle mass)^13^. Muscle strength was estimated based on hand grip strength, evaluated by digital manual dynamometry (Jamar Digital Plus + Hand Dynamometer and Simmons Preston, Canada). Cut-off points for loss of strength were < 30 kg for men and < 20 kg for women^14^. Finally, the muscular performance was evaluated by the 4 meter gait speed test, considering as “loss” a path velocity < 0.8 m/s^14^. The combination of these tests allowed the categorization of individuals into sarcopenic (presence of loss of muscle mass associated with loss of muscle strength or performance) or non-sarcopenic. The detailed methodology of collecting the above data can be verified in a previous article originating from the same study^13^.

The use of medication was evaluated in the 15 days prior to the interview. To better qualify the information, respondents were asked to show the packaging or prescription of the medicines used. We carried out a classification by pharmacological groups according to the ATC classification (Anatomical Therapeutic Chemical Classification System) recommended by WHO^15^. Among the drugs, we classified those potentially related to the occurrence of falls among the elderly: psychoanalytics, psycholeptics, antiepileptics, calcium channel blockers, diuretics, muscle relaxants and, among the medications used for cardiac therapy, digoxin.

Functional ability was assessed from basic activities (eating, bathing, dressing, using the toilet, lying down and getting up from the bed or chair, and controlling the functions of urinating or evacuating) using the Katz index^16^ and instrumental activities (using the telephone, going to distant places using transportation, shopping, tidying up, washing clothes, preparing meals, taking medication, and taking care of money) measured by the Lawton scale^17^. For both instruments, the participants were classified as independent (did not need help to perform any activity) or dependent (they needed partial or total help to perform at least one activity).

Regarding the behavioral characteristics, self-reported alcohol dependence (measured by the CAGE method)^18^ was evaluated positive for the person’s perception of their dependence on alcoholic beverages (up to one positive answer = negative; two or more positive answers = positive) and the level of physical activity, measured by the International Physical Activity Questionnaire (IPAQ)^19^, classified as active those who reported practicing ≥ 150 weekly minutes of physical activities in leisure and travel.

The gross and adjusted prevalence ratios were obtained by means of Poisson regression, with robust adjustment for the variance, with the calculation of the p-value for heterogeneity or linear trend for ordinal variables. The adjusted analysis was performed respecting the hierarchical levels: in the first, the demographic, socioeconomic and occupational variables (sex, age, marital status, education, economy class, work situation) were added; in the second, the behavioral and health-related variables (physical activity level, self-reported alcohol dependence, sarcopenia and morbidity score) were added; in the third level, we added the drugs that could potentially cause falls and in the fourth and last level we added the variables of disabilities. The variables were adjusted to each other within each level and for the higher level. Those with p ≤ 0.20 were maintained in the model for confounding control and those with p < 0.05 were considered to be associated with the outcome. Due to the complex design, the analyses were weighted considering the census tract (cluster). For the Poisson regression analysis, the svy command was used to correct for complex sampling.

The analyses were conducted in the Stata 12.1® program.

The research was approved by the Research Ethics Committee of the Faculdade de Medicina of the Universidade Federal de Pelotas (Protocol 472.357/2013). The study participants signed the free and informed consent form.

## RESULTS

We identified 1,844 eligible older adults and, after successive contact attempts, 393 losses and refusals were recorded (21.3%). Of the set of 1,451 elderly interviewed, 1,448 presented complete information on the evaluated outcome, constituting the final sample of this study.

The majority of the older adults were female (63.0%) and about 50.0% were between 60 and 69 years old. Approximately 53.0% of the interviewees reported having a partner and 56.8% were of economy class C. Regarding education, 31.0% had four to seven years of schooling and 80.4% were not employed in the interview period. The prevalence of three or more morbidities was 38.0% and sarcopenia approximately 14.0%. Only 1.0% of the elderly had alcohol dependence, about 60.0% were insufficiently active in leisure and travel. Almost 70.0% of the elderly were using some medication that could cause falls, and about 35.0% were dependent for basic and instrumental activities of daily living (Table 1).

**Table 1:** Description of the sample according to demographic, socioeconomic, behavioral and health variables. Pelotas, state of Rio Grande do Sul, 2014. (n = 1.448)

Variable	n	%
Gender		
Male	537	37.0
Female	914	63.0
Age (in complete years)		
60–69	756	52.3
70–79	460	31.8
80 or older	230	15.9
Marital status		
With partner	763	52.7
Without partner	684	47.3
Economic class (ABEP)		
A/B	384	27.9
C	781	56.8
D/E	201	15.3
Schooling (full years of study)		
None	196	13.6
1–3	337	23.4
4–7	445	31.0
8–11	143	10.0
12 or older	316	22.0
Currently employed		
No	1,084	80.4
Yes	264	19.6
Morbidity^a^		
None	174	12.2
1	348	24.3
2	366	25.5
3 or more	545	38.0
Sarcopenia		
No	1,112	86.1
Yes	179	13.9
Self-reported alcohol dependence (CAGE)		
No	1,436	99.0
Yes	15	1.0
Use of drugs that may cause falls^b^		
No	409	31.3
Yes	896	68.7
Physical activity level (IPAQ)		
Insufficiently active	824	60.1
Active	548	39.9
Functional capacity (Katz)		
Independent	920	63.9
Dependent	520	36.1
Functional capacity (Lawton)^c^		
Independent	837	66.0
Dependent	432	34.0

ABEP: *Associação Brasileira de Empresas de Pesquisas*; CAGE: Cut down, Annoyed by criticism, Guilty and Eye-opener; IPAQ: International Physical Activity Questionnaire

^a^ Hypertension, diabetes, heart problem, stroke, arthritis, osteoporosis, Parkinson’s disease, chronic kidney failure, glaucoma, emphysema.

^b^ Psychoanalytics, psycholeptics, antiepileptics, calcium channel blockers, diuretics, muscle relaxants and digoxin.

^c^ Higher number of missing: 182.

The prevalence of falls in the last year was 28.1% (95%CI 25.9–30.5). Regarding the number of falls, 51.5% (95%CI 46.6–56.4) of the older adults suffered a single fall, 25.2% (95%CI 21.0–29.5) fell twice and 23.3% (95%CI 19.1–27.4) fell three or more times in the last year (data not shown in the table). Most of the participants who fell had fallen at home (56.4%) or in the street (46.6%) (Table 2). As a consequence of the fall, 12.1% (CI95% 8.9–15.3) reported having fractured some bone (data not shown), and the most affected sites were the lower limbs (4.9%) and the upper limbs (3.9%).

**Table 2:** Description of the location of the falls and occurrence of fractures among older adults living in Pelotas, state of Rio Grande do Sul, 2014.

Variable	Total elderly			Elderly who fell down		
	n	%	95%CI	n	%	95%CI
Fall location						
House/Patio	230	15.9	14.0–17.7	230	56.4	51.5–61.2
Street	190	13.1	11.4–14.8	190	46.6	41.7–51.4
Another person’s house/Patio	23	1.6	9.4–2.2	23	5.6	3.4–7.9
Another location	31	2.1	1.4–2.9	31	7.6	5.0–10.2
Fractured bone						
Upper limbs	16	1.1	0.6–1.6	16	3.9	2.0–5.8
Torso	9	1.4	0.8–2.0	9	2.2	0.8–3.6
Hip	2	0.1	0.0–0.3	2	0.5	0.2–1.2
Lower limbs	20	0.6	0.2–1.0	20	4.9	2.8–7.0
Other	1	0.1	0.0–2.0	1	0.3	0.2–0.7

Self-reported alcohol dependence and drug use were not significantly associated with the outcome in the gross analysis. In the adjusted analysis, the occurrence of falls among older adults was associated with gender, age, economic class, schooling, current work, morbidity and functional capacity by the Lawton scale. The women presented a prevalence of falls almost 1.5 times greater when compared to the men. Regarding age, a linear trend was observed, and the highest prevalence of falls was among those aged 80 years or older (PR = 1.27, 95%CI 0.97–1.66), when compared with those aged 60 to 69 years old. Older adults in the C and D/E classes had a higher prevalence of falls when compared to those in the A/B economic class, with a linear trend. For schooling, a linear trend was also observed, and older adults with no schooling presented a 47.0% greater prevalence of falls when compared to those with a high schooling level. The prevalence of falls was 50.0% lower among older adults with a chronic disease when compared to those without chronic disease. However, protection decreased with increasing numbers of chronic diseases. Older adults who were not employed had 30.0% greater prevalence of falls compared to those who were working. Older adults who were dependent for instrumental activities of daily living (Lawton) also had a higher prevalence of falls compared to independent participants (PR = 1.38, 95%CI 1.10–1.73) (Table 3).

**Table 3:** Factors associated with the occurrence of falls by the elderly. Pelotas, state of Rio Grande do Sul, 2014. (n = 1,448)

Level	Variable	Gross PR	95%CI	p	Adjusted PR	95%CI	p
1	Gender			< 0.001			< 0.001
	Male		1.00			1.00	
	Female	1.57	1.30–1.90		1.49	1.22–1.82	
1	Age (in complete years)			< 0.001^a^			0.040^a^
	60–69		1.00			1.00	
	70–79	1.32	1.07–1.61		1.23	0.99–1.52	
	80 or older	1.50	1.19–1.88		1.27	0.97–1.66	
1	Marital status			0.001			0.380
	With partner		1.00			1.00	
	Without partner	1.37	1.14–1.65		1.10	0.89–1.37	
1	Economic class (ABEP)			0.001^a^			0.017^a^
	A/B		1.00			1.00	
	C	1.33	1.05–1.69		1.29	1.01–1.65	
	D/E	1.62	1.22–2.14		1.45	1.06–1.99	
1	Schooling (full years of study)			0.001^a^			0.02^a^
	None	1.70	1.22–2.39		1.47	1.08–1.98	
	1–3	1.36	1.00–1.87		1.30	0.98–1.72	
	4–7	1.42	1.06–1.91		1.40	1.09–1.80	
	8–11	1.31	0.88–1.94		1.35	0.94–1.94	
	12 or older		1.00			1.00	
1	Currently employed			0.001			0.048
	No	1.60	1.23–2.09		1.33	1.00–1.76	
	Yes		1.00			1.00	
2	Morbidity^b^			< 0.001			0.002
	None		1.00			1.00	
	1	0.69	0.49–0.97		0.48	0.32–0.72	
	2	0.94	0.69–1.28		0.63	0.43–0.90	
	3 or more	1.44	1.09–1.89		0.89	0.62–1.26	
2	Sarcopenia			0.016			0.270
	No		1.00			1.00	
	Yes	1.34	1.06–1.70		1.18	0.88–1.57	
2	Alcohol dependence (CAGE)			0.682			0.905
	No		1.00			1.00	
	Yes	1.19	0.52–2.69		0.92	0.23–3.74	
2	Physical activity level (IPAQ)			0.003			0.918
	Insufficiently active	1.31	1.10–1.56		0.99	0.80–1.23	
	Active		1.00			1.00	
3	Medication use^c^			0.121			0.979
	No		1.00			1.00	
	Yes	1.16	0.96–1.41		1.00	0.76–1.32	
4	Functional capacity (Katz)			< 0.001			0.307
	Independent		1.00			1.00	
	Dependent	1.70	1.47–1.98		1.13	0.89–1.43	
4	Functional capacity (Lawton)^d^			< 0.001			0.006
	Independent		1.00			1.00	
	Dependent	1.81	1.49–2.20		1.38	1.10–1.73	

ABEP: *Associação Brasileira de Empresas de Pesquisas*; CAGE: Cut down, Annoyed by criticism, Guilty and Eye-opener; IPAQ: International Physical Activity Questionnaire

^a^ linear trend test.

^b^ Hypertension, diabetes, heart problem, stroke, arthritis, osteoporosis, Parkinson’s disease, chronic kidney failure, glaucoma, emphysema.

^c^ Psychoanalytics, psycholeptics, antiepileptics, calcium channel blockers, diuretics, muscle relaxants and digoxin.

^d^ Higher number of missing: 182.

Diabetes, heart disease, stroke, arthritis, osteoporosis and Parkinson’s disease were associated with the occurrence of the outcome in the gross analysis. Associations with osteoporosis, sarcopenia and Parkinson’s disease lost statistical significance in the adjusted analysis. The prevalence of falls was higher among older adults with diabetes (PR = 1.24, 95%CI 1.03–1.50), heart problem (PR = 1.24, 95%CI 1.02–1.51), who had a stroke (PR = 1.44, 95%CI 1.14–1.81) and had arthritis (PR = 1.25, 95%CI 1.06–1.48), when compared to those without these diseases (Table 4).

**Table 4:** Main health problems related to the occurrence of falls by the elderly. Pelotas, state of Rio Grande do Sul, 2014. (n = 1,448)

Morbidity	Gross PR	95%CI	p	Adjusted PR*	95%CI	p
Diabetes			< 0.001			0.023
No		1.00			1.00	
Yes	1.37	1.16–1.63		1.24	1.03–1.50	
Hypertension			0.633			0.082
No		1.00			1.00	
Yes	1.04	0.88–1.23		0.85	0.71–1.02	
Heart problem			< 0.001			0.029
No		1.00			1.00	
Yes	1.46	1.23–1.74		1.24	1.02–1.51	
Stroke			< 0.001			0.002
No		1.00			1.00	
Yes	1.49	1.20–1.85		1.44	1.14–1.81	
Arthritis			< 0.001			0.010
No		1.00			1.00	
Yes	1.51	1.27–1.78		1.25	1.06–1.48	
Osteoporosis			< 0.001			0.392
No		1.00			1.00	
Yes	1.50	1.27–1.77		1.08	0.90–1.30	
Parkinson’s disease			0.039			0.283
No		1.00			1.00	
Yes	1.64	1.03–2.62		1.35	0.78–2.33	
Renal failure			0.949			0.703
No		1.00			1.00	
Yes	0.99	0.65–1.49		0.92	0.58–1.44	
Glaucoma			0.207			0.896
No		1.00			1.00	
Yes	1.21	0.90–1.64		0.98	0.73–1.32	
Pulmonary emphysema			0.244			0.515
No		1.00			1.00	
Yes	1.19	0.88–1.61		1.10	0.82–1.48	

* Adjustment for demographic and socioeconomic variables and other chronic diseases of the model.

## DISCUSSION

One in three older adults suffered at least one fall in the last year and 12.0% of them fractured some bone as a result of that fall. The injury occurred mainly in their own residence. The prevalence of falls was higher in women, individuals of more advanced age, lower educational level and economic class, who were not employed and who presented functional incapacity for instrumental activities. The prevalence of falls was also higher in patients with diabetes, heart disease, arthritis and those who reported having had a stroke.

The prevalence of falls in the last year found in this study (28.1%) is similar to that found in other Brazilian studies, whose prevalence ranges from 28% to 37.5%^10,20–22^, as well as those found in studies conducted in countries in Latin America and the Caribbean, where prevalence ranged from 21.0% to 34.0%^23^ ^,^ ^24^. Differences in prevalence among studies should be interpreted with caution, since they may be due to the delineation of studies and the methodologies adopted or because they are point estimates that have error margins^25^.

As in other studies, the majority of the sample was female^10,25–29^, which can be explained by the fact that women live longer than men^30^. In addition, the prevalence of falls in this and other studies was higher among women^10,25–29^, but the mechanisms underlying this association are still unclear. Some factors pointed out by other authors concern the difference in the body composition of the women when compared to the men. Women have lean mass and muscle strength in smaller amounts and greater loss of bone mass due to the reduction of estrogen levels. This increases the likelihood of osteoporosis in this group^25^ ^,^ ^29^ ^,^ ^31^ and, consequently, the risk of falls.

Age was positively associated with the outcome, which confirms findings in the national and international literature^10,25,32–34^. This relationship occurs because the biological aging process involves structural and functional changes, such as decreased muscle strength and elasticity, impairment of joint stability and dynamics, and changes in the sensory and nervous system. Such changes compromise postural control and are capable of altering gait and balance, culminating, consequently, in an elevated risk of this outcome^28^ ^,^ ^35^.

Economic class and schooling were inversely associated with the risk of falls, an association also found in other studies^10^ ^,^ ^36^. A possible explanation for this association would be the greater difficulty of individuals with low socioeconomic status to access health services and consequently prevent factors that could lead to falls^36^.

The prevalence of falls was higher in unemployed older adults. The literature shows an inverse association between income and risk of falls^6,36–38^. It is likely that employed older adults will have a better financial situation, as well as being healthier, as they are fit for work and therefore less likely to fall^6^.

Physical activity is indicated for the prevention of falls among older adults, since certain activities, such as those involving strength and balance, promote increased muscle and bone strength, coordination, walking speed, functional ability and quality of life^39^. However, the present study did not observe an independent association, perhaps because it did not distinguish the types of activity performed by the participants, presenting only whether they were active or not.

Likewise, there was no statistically significant association between falls and sarcopenia after adjustment for several factors, including gender. Baumgartner, in 1998, when stratifying the occurrence of falls by gender, found an increased risk for falls among sarcopenic older adults, but this association was also not statistically significant for females^40^. Nevertheless, even when the analysis was stratified by gender, no association was found between sarcopenia and occurrence of falls (data not shown in the table). Muscular weakness is cited as one of the risk factors for falls in a guideline drawn up by different medical societies^41^ and corroborated by Sayer in an analysis of the Hertfordshire cohort^42^. However, muscle weakness is not a necessary or sufficient criterion for sarcopenia – only a collaborator. Perhaps the combination with the other diagnostic factors for the syndrome (loss of muscle mass and muscular performance) influences this association to the point of taking away the statistical significance. Therefore, although theoretically the association between falls and sarcopenia was plausible, it was not verified in this study.

The occurrence of falls was associated with functional capacity, assessed through instrumental activities (Lawton scale), while the association was not observed for basic activities (Katz scale). This result may be due to the fact that basic activities are more closely related to personal care and instrumental activities assess the ability of the older adults to perform activities to manage their life and home independently. Some of these activities involve getting around (using transportation, shopping, tidying up, washing clothes), and failure to perform them efficiently can increase the risk of falls. Perracini et al. report that older adults with difficulty to perform between one and three activities of daily living have 2.37 times more chance of suffering falls^27^. However, the relationship between falls and functional disability is subject to reverse causality bias. Falls among older adults can result in functional disability, either due to the occurrence of fractures that make it impossible to perform the activities or the fear of suffering a new fall^43^. Older adults, with functional limitations that hamper daily activities, which may occur due to lack of balance or motor coordination, are at increased risk of falls.

Elderly people with arthritis, depressive symptoms, orthostatic hypotension, and cognitive, visual, balance, gait, or muscular strength deficits are at increased risk for falls^44^ ^,^ ^45^. As for diabetes, this relationship is well established in several studies^46^ ^,^ ^47^, with complications due to peripheral neuropathy, reduced vision and decreased renal function as the main causes. Balance, strength, and gait issues are likely intermediate factors in any association between diabetes complications and increased risk of falls^46^. Strokes may lead to hemiplegia or paresis of the lower limbs, affecting the gait of the individual, who assumes an unstable erect position with an impaired support base. As a consequence, visual dysfunction and spatial-visual injury may also occur, thus influencing the older adult’s balance and mobility^47^. In relation to heart problems, certain arrhythmias, such as atrioventricular blocks, sinus node changes and bradycardia may also cause falls^48^. In addition, the fall may be the first sign of an acute asymptomatic myocardial infarction^49^.

Regarding the use of medication considered to potentially cause falls, this study found no association, although several studies have demonstrated the plausibility of this relationship^50–53^. However, in this study, only drugs that could potentially cause falls were evaluated, whereas in studies that show an association, any type of medication is considered, which could explain in part the divergence in the results.

This study has limitations. The prevalence of falls found may be underestimated, because, due to survival bias, older adults who suffered falls and who had more serious health complications caused by the fall may already have died. The memory bias is also not discarded, considering the study population. However, hopefully this was not such a big problem since factors such as the occurrence of falls tend to be strongly recalled because of the impact they usually have on health. As strengths of the study, it is worth mentioning the quality of the sample method, which allowed the analysis of a representative sample of older adults from the urban area of Pelotas. In addition, quality control was performed in the various stages of the study, such as interviewer training, instrument testing, data quality control, and ensuring greater credibility of the analyzed data. The study was also able to evaluate a series of factors that, according to previous literature, could be related to the occurrence of falls, allowing us to draw a profile of characteristics predisposing to the occurrence of this injury.

The high prevalence of falls among older adults makes it clear that steps should be taken to prevent them. Prevention strategies should be focused on vulnerable groups, i.e. at higher risk for falls, such as older people with low socioeconomic status who are not working and are dependent for functional capacity.
